# Temperature and Pollinators Drive Evolutionary and Plastic Variation in Leaf and Petal Epicuticular Waxes

**DOI:** 10.1007/s10886-026-01691-w

**Published:** 2026-02-13

**Authors:** Corinne Hertaeg, Florian P. Schiestl

**Affiliations:** Department of Systematic and Evolutionary Botany, University of Zürich, Zürich, Switzerland

**Keywords:** Epicuticular wax composition, Chemical ecology, Plant-pollinator interactions, Herbivory, Thermal adaptation, *Brassica rapa*

## Abstract

Plants rely on epicuticular waxes to withstand stress and mediate interactions with other organisms, yet floral waxes remain largely unexplored despite their potential key role in reproduction. We examined how temperature and pollinators shaped leaf and petal waxes in *Brassica rapa* after six generations of experimental evolution under hot and ambient conditions with bumblebees (*Bombus terrestris*), *Pieris rapae* butterflies, acting as “pollinating herbivores” and also involving larval herbivory, or hand-pollination as a control. Leaves contained more wax but shorter aliphatic chains than petals. Heat reduced leaf waxes yet elongated chains, while petals lost wax and shortened chains, revealing opposite responses across organs. Insect-pollinated plants, especially those pollinated by bumblebees, showed reduced heat-driven wax loss in petals, mitigated reductions in chain length or even increases, and altered potential signalling compounds such as methyl-alkanes and α/β-amyrins, while in leaves exhibiting reduced wax amounts and dampened increases in chain-length. *P. rapae* exposure increased or maintained high levels of compounds linked to herbivore defence (long alcohols, indole-3-acetonitrile), suggesting possible herbivore-specific responses. These results uncover a leaf–petal wax trade-off and identify pollinators as key drivers of wax chemistry under heat. We show that leaf and petal waxes are both plastic and evolvable, emphasizing floral waxes as integral components at the crossroads of stress tolerance, herbivory, and pollination.

**Supplementary Information:**

The online version contains supplementary material available at https://doi.org/10.1007/s10886-026-01691-w.

## Introduction

To reduce water loss and cope with environmental stresses, plants evolved a protective layer crucial for their survival and ecological success: the plant cuticle. While the cutin matrix provides mechanical strength (Jeffree 1996), cuticular waxes (Jetter et al. 2006) offer waterproofing (Riederer and Schreiber 2001) and protection from UV, drought, salinity, and temperature (Shepherd and Griffiths 2006; Pfündel et al. 2008; Lewandowska et al. 2020). Beyond abiotic protection, these waxes also mediate biotic interactions with herbivores and pathogens (Eigenbrode and Espelie 1995; Müller and Riederer 2005). Leaf waxes, due to their importance in water retention and pest resistance, are well-characterized, yet wax composition varies across plant organs (Hennig et al. 1988; Buschhaus et al. 2015; Guo and Jetter 2017; Guo et al. 2017; Cheng et al. 2019, 2021; Zhao et al. 2019), reflecting organ-specific selective pressures. Floral waxes, which shape flower surface properties (Whitney et al. 2009; Glover and Whitney 2010; Buschhaus et al. 2015; Cheng et al. 2021; Liao et al. 2021), likely affect reproduction, yet they remain surprisingly understudied, leaving a major gap in our understanding of how flowers chemically and physically interact with their environment, including pests and pollinators.

Flowers are essential for plant fitness, yet compared to leaves they are short-lived, fragile, and prone to water loss (Roddy et al. 2023). Most flowers have less wax than leaves (but see: Guo and Jetter 2017; Guo et al. 2017; Cheng et al. 2019, 2021) and often lack the wax crystals commonly found on leaf surfaces (Tunstad et al. 2024). This may reflect a cost-optimization strategy, as investing in thick, protective flower cuticles may be excessive for such short-lived structures (Roddy et al. 2023). Moreover, thick cuticles could interfere with a flower’s primary function: attracting pollinators. Flower cuticle waxes influence several traits critical to pollinator attraction. For instance, volatile emission depends on diffusion through the cuticle (Liao et al. 2021), which thick layers can inhibit; wax crystals may reduce pollinator grip and hinder handling (Whitney et al. 2009); surface structure affects optical properties including colour and iridescence (Glover and Whitney 2010); and cuticle permeability likely regulates floral humidity (Buschhaus et al. 2015; Cheng et al. 2021), a key sensory cue for bumblebees and hawkmoths (Von Arx et al. 2012; Harrap et al. 2021). These trade-offs raise the question of whether plants adapt their floral epicuticle waxes, the outermost wax layer, to environmental conditions and the preferences of their primary pollinators and herbivores.

While pollinator responses to visual (van der Kooi et al. 2019) and olfactory (Raguso 2008) floral signals are well documented, the effects of other signals and cues, such as waxy epicuticular compounds, remain poorly understood, even though many pollinators inevitably contact the surface microstructure and chemical composition of flower cuticles (Moyroud and Glover 2017). The chemical wax composition may serve as short-range recognition signals to more reliably identify flowers and their quality or as indicators of previous visitors (Yokoi et al. 2007; Witjes and Eltz 2009). Floral scent studies suggest that chemicals attracting or repelling insects may have evolved from pre-existing signals in insect communication (Schiestl 2010). Similarly, pollinators likely use preexisting receptors for insect cuticular compounds, which are chemically surprisingly similar to plant cuticles (Hadley 1981), to perceive plant epicuticular waxes. Alkanes, common components of floral waxes (Tunstad et al. 2024), serve as fertility signals and nest recognition cues in bumblebees (Sramkova et al. 2008; Rottler et al. 2013), mate discrimination signals in *Papilio polytes* butterflies (Ômura et al. 2020), and, in combination with alkenes, as sex pheromones in some flies (Ferveur 1997; Liu et al. 2025). This suggests that many pollinators have sophisticated systems for detecting floral epicuticular wax compounds.

The best-documented example of floral wax involvement in pollination are sexually deceptive orchids, which mimic female insect cuticles and emit sex pheromones to attract male pollinators attempting to copulate (Schiestl et al. 1999). While orchid cuticle chemistry is shaped by pollinator-driven selection, it is unknown if other plants use similar chemical signals and how generalist pollinators respond. However, bees, for example, can distinguish cuticular compounds by carbon chain length and functional groups (Guerrieri et al. 2005), suggesting they are highly sensitive to compounds on flower epicuticles (Schiestl and Johnson 2013). While it is in the plant’s best interest to attract bees and present them with appealing flowers, pollinating herbivores pose a more complex challenge. Lepidopterans, whose larvae feed and develop on host plant tissue, represent a double-edged sword, making trade-offs between butterfly attraction and repellence likely. Because lepidopterans act as pollinating herbivores that interact with both flowers and leaves, we expect them to shape the evolution of epicuticular wax in both tissues in plants for which they are the primary pollinators and herbivores.

Herbivores and plant epicuticular waxes interact in multiple ways (Eigenbrode and Espelie 1995; Müller and Riederer 2005). Wax microstructure can affect visual cues important for host plant detection (Prokopy et al. 1983) as well as herbivore attachment and locomotion, with higher wax amounts and crystals generally reducing attachment forces (Stork 1980; Gorb and Gorb 2017). Additionally, some insects use compounds from the wax layer as host plant selection cues (Adati and Matsuda 1993; Lin et al. 1998; Powell et al. 1999; Morris et al. 2000; Coyle et al. 2003), suggesting that wax removal may reduce herbivore attacks. Consistent with this, reduced wax amounts in so-called glossy phenotypes have often been associated with lower susceptibility to insect herbivory (Eigenbrode and Espelie 1995). However, these effects are context dependent: glossy phenotypes can deter some pests, including some lepidopterans (Dickson et al. 1990; Stoner 1990), while increasing susceptibility to others such as aphids and flea beetles (Way and Murdie 1965; Stoner 1990; Bodnaryk 1992; Lamb et al. 1993). Such context-dependent effects may explain why some plants, including *Beta vulgaris*, apple, and wheat, adjust leaf wax amounts or composition in response to herbivory (Bystrom et al. 1968; Dutton et al. 2002; Kosma et al. 2010). Notably, while these responses have been studied almost exclusively in leaves, herbivore-induced changes in floral waxes remain largely unexplored.

In addition to biotic factors, abiotic conditions might also influence floral wax amounts and composition. In *Brassica* species, leaf wax amounts increase under lower temperatures, and the relative abundance of compound classes shifts with changes in light intensity and temperature (Whitecross and Armstrong 1972; Baker 1974; Armstrong and Whitecross 1976; Reed and Tukey 1982). However, it remains unknown whether floral waxes exhibit similar responses to environmental stresses. It is also unclear whether the observed plastic changes in leaf waxes are adaptive, and whether they could lead to evolved differences in wax quantity and composition under sustained elevated temperatures. The responses of floral waxes are even less well understood. To address these questions, we investigated how floral and leaf waxes respond to elevated temperatures predicted under climate change (Fischer et al. 2022; MétéoSuisse 2024) and how pollinators and pollinating herbivores influence these wax profiles, using plants that underwent six generations of experimental evolution with different pollination and herbivory regimes, under ambient and hot conditions.

We analysed evolved and plastic responses of leaf and petal epicuticular waxes of fast-cycling *Brassica rapa* plants that evolved under different conditions (Traine et al. 2024). During the experimental evolution part, plants either experienced ambient (22 °C) or hot (27 °C, plus 30 °C “heatwave” for 24 h once per week) temperatures and were either pollinated by bumblebees (*Bombus terrestris*), butterflies (*Pieris rapae*) or hand pollinated. Insect-pollinated plants experienced selection for more efficient pollination, and *P. rapae*-pollinated plants additionally faced caterpillar herbivory proportional to eggs received during butterfly pollination. *P. rapae* is known to be as efficient a pollinator as bees but visits less often (Sahli and Conner 2007; Rader et al. 2009), which was accounted for in the pollination regime. Hand-pollination served as a control for greenhouse conditions with no pollinator selection. We analysed leaf and petal waxes in an additional generation grown under ambinet (22 °C) and hot (27 °C, 30 °C for 24 h a week) conditions. This allowed us to assess both plastic responses and evolutionary changes in wax composition and amount in response to temperature, pollinator, and pollinating herbivore treatments. Based on previous studies, we expect petals to have less wax, shorter compounds, and distinct composition compared to leaves. In *Brassica*, only plastic leaf wax changes in response to temperature have been reported (Whitecross and Armstrong 1972; Baker 1974; Armstrong and Whitecross 1976; Reed and Tukey 1982), but we anticipate evolved changes as well. We further hypothesize that floral waxes, like leaf waxes, can plastically change and evolve in response to temperature, and that petal waxes may additionally evolve under pollinator selection: bumblebee-pollinated plants are expected to evolve more attractive petal waxes, while *P. rapae*-pollinated plants may increase floral attractiveness for adults (pollinators) while reducing leaf attractiveness for oviposition and larval feeding.

## Methods and Materials

### Plants and Experimental Design

This study has two components: an experimental evolution phase to assess evolved differences, followed by a greenhouse experiment under two different temperature conditions to assess plastic responses to altered temperature. We used rapid-cycling *Brassica rapa* L. plants (Wisconsin Fast Plants™), which were the outcome of the evolution experiment conducted between 2021 and 2023 (Traine 2025; Traine et al. 2025). *B. rapa* is an annual, mostly self-incompatible species; the used variety has a short generation time (~ 35 days) and high genetic variance, making it well-suited for experimental evolution and selection studies (Gervasi and Schiestl 2017; Ramos and Schiestl 2019; Traine et al. 2024). Over six generations, the ancestral lines of the plants used for the second phase were exposed to either ambient (22 °C) or hot (27 °C with 24 h 30 °C per week) temperature regimes and evolved under different pollinator treatments: *Bombus terrestris* bumblebees as a bee pollinator, *Pieris rapae* butterflies as a pollinating herbivore, and hand pollination.

Insect-pollinated plants may have experienced selection for better performance in the presence of their respective pollinators, as the number of offspring in the next generation depended on seed production. In contrast, hand-pollinated plants did not experience pollinator-mediated selection and served as a control for background effects (e.g., germination, seed mortality) under greenhouse conditions. These plants were pollinated with a mixed pollen pool from all controls, with each individual contributing one seed to the next generation, likely preserving the genetic diversity of generation one. To mimic natural conditions, butterfly-pollinated plants were exposed to caterpillar herbivory. The number of caterpillars per plant was determined by the number of eggs deposited, categorized into five groups representing 0–4 caterpillars. Since *P. rapae* butterflies are efficient pollinators but visit less often than bees (Sahli and Conner 2007; Rader et al. 2009), the pollination regimes were adapted accordingly. Twelve sequentially released bees were allowed to visit 4 plants each, which resulted in 3 pollinated plants per bee (20–27 plants out of a population of 49) that contributed to the next generation proportionally to the number of seeds produced. Since butterflies visited less frequently, 25 of them were released in a cage with all 49 plants for 2 days and each of these plants contributed to the next generation proportionally to the number of seeds they produced. This experiment assessed evolved plant responses to increased temperature and different pollinator treatments. Each treatment was replicated so that evolutionary replicates A and B experienced the same conditions (same greenhouse at the same time) and pollination regimes but evolved independently, allowing us to account for random changes (drift) within treatments.

Plants used in the second stage, which assessed plastic responses to different growing temperatures, were grown from seeds produced by generation seven of the evolution experiment. To minimize maternal effects, all generation-seven plants (from all treatments) were grown under the same temperature, humidity, and soil conditions as the ambient genotypes described below. They were hand-pollinated within treatments using the same random procedure as for controls and had no exposure to pollinators or herbivores. For the plasticity experiment, we used seeds of twelve generation-seven individuals per treatment (both temperatures and all three pollination regimes) and evolutionary replicate (A and B), sowed them in 54-well plates (sowing soil with equal parts sterilized sieved topsoil, compost, pumice (1–5 mm) and peat) and allowed them to germinate for seven days in a phytotron (22 °C, 60% RH, 24 h light). Then plants were re-potted in individual 7 × 7 × 8 cm pots filled with standardized soil (Einheitserde, Germany) and placed into one of two greenhouse chambers. The ambient chamber was set to 22 °C and the hot one to 27 °C with one heat day of 30 °C per week (60% RH,16 h light, 8 h dark). Ambient genotypes (plants evolved under ambient conditions) were exposed to both ambient and hot temperatures, whereas hot genotypes (evolved under hot conditions) were only exposed to hot temperatures (Table 1). As a result, plastic responses could be assessed only in ambient genotypes, reflecting our focus on plant responses to mid-century early summer temperatures projected for northern Switzerland (Fischer et al. 2022; MétéoSuisse 2024).

**Table 1:** Experimental design. Plants evolved in hot conditions were only grown in the hot greenhouse. To avoid pseudo replication, each of the 12 plants per treatment and evolutionary replicate is the offspring of a different plant from the evolution experiment. Hand pollination refers to our control treatment

Evolved temperature	Pollinator treatment	Ambient greenhouse		Hot greenhouse	
		Replicate A	Replicate B	Replicate A	Replicate B
Ambient	Hand	12	12	12	12
	Bee	12	12	12	12
	*Pieris*	12	12	12	12
Hot	Hand	-	-	12	12
	Bee	-	-	12	12
	*Pieris*	-	-	12	12

### Tissue Sampling and Chemical Analysis

As soon as plants had ten freshly opened flowers simultaneously (23–35 days after sowing), we sampled and pooled the petals of these 10 flowers in 4 ml amber glass vials (Agilent technologies, storage vials, closed top with PTFE/silicone septa). If 10 fresh flowers were never reached, we sampled as many as possible 35 days after sowing. We collected petals in the morning and early afternoon and extracted the same day in 1 ml chloroform (Sigma Aldrich, 99,8% + stab. EtOH) with 5 µg Octadecane as an internal standard, for 1 min. Simultaneously, we collected the first real leaf of each plant, which we briefly flattened under a glass pane, photographed for area measurement using ImageJ, and extracted in 1.5 ml chloroform with 5 µg internal standard. Extracts were stored in 2 ml vials (Agilent A-Line, clear, screw-top) at -20 °C until further processing. To estimate petal area, one petal from each of two additional flowers per plant was mounted on white paper with 4 mm grid lines using clear tape, photographed, and measured in ImageJ; total petal area was then calculated by multiplying by the number of sampled petals. Petal and leaf area was later used to express wax amounts per unit surface area (µg/cm²), allowing a direct comparison of wax quantities between leaves and petals despite their differences in size.

We prepared the wax extracts for GC analysis by drying 250 µl of each sample under a gentle N_2_ flow, then dissolving in 30 µl BSTFA (bis-N, O-trimethylsilyltrifluoroacetamide, UCT, Selectra-Sil, BSTFA) and 30 µl pyridine (Scharlab, ultrapure (≥ 99.9%)). The mixture was incubated at 70 °C for 1 h, after which we evaporated excess reagents under N_2_ and added 200 µl chloroform. We analysed each sample on a GC-FID system to quantify the compounds and made pools of 6 samples to run on a GC-MSD system to identify compounds. 1 µl of each sample was injected into the GC (Agilent 6890 N; Agilent Technologies, Palo Alto, CA, USA) equipped with a HP-5 capillary column (Agilent, 15 m length, 0.25 mm diameter, 0.25 μm film thickness). For FID analysis we used hydrogen at a constant flow of 1.5 ml/min, and for the MSD analysis helium at a constant flow of 2 ml/min as carrier gas. We set the inlet temperature to 250 °C and the injector to splitless mode. The oven program started at 40 °C (1 min), ramped at 4 °C/min to 80 °C, then at 10 °C/min to 300 °C, held for 5 min. An Agilent 5975 MSD mass spectrometer was used for compound identification. We identified compounds using a C7-C40 alkane standard, Kovat’s retention indices, characteristic ions, comparison to synthesized standards and the NIST (the National Institute of Standards and Technology) library. Some compounds could not be identified and are listed as unknowns with their retention times, while others could only be classified by compound class, which is indicated together with the retention time. For compound quantification we used the CHEMSTATION Enhanced data analysis software (v.E.01.00, Agilent Technologies AG, Santa Clara, CA, USA).

### Statistical Analysis

All statistical analyses were performed in R version 4.4.1. (R Core Team 2025). We pooled data from evolutionary replicates A and B since they were not significantly different from each other. We first analysed total wax amounts. Since the assumption of normally distributed differences between wax amounts on petals and leaves were not met, we used a paired *Wilcoxon signed-rank test* to compare wax amounts on leaves and petals. Within the petal data, a *Levene’s test* showed heteroscedasticity across hot and ambient grown and evolved plants and within leaf data across pollinator treatments. We therefore used gls models (linear model using generalized least squares, package: nlme, gls function) and applied a variance structure (varIdent) that allowed residual variances to differ by pollinator or temperature treatment. In the following steps we analysed leaf and petal data separately. In a first model, we tested the effect of growing temperature, pollinator and their interaction on wax amounts and in the second the effect of evolved temperature, pollinator and their interaction.

Next, we analysed the length of aliphatic compounds. We converted each compound’s peak area to its proportion of the total peak area for each petal and leaf sample. Then, we calculated an average aliphatic compound length using the weighted arithmetic mean formula. Since petals and leaves had different sets of compounds, we calculated separate means and analysed the data separately. To account for the heteroscedasticity across groups (growing temperature, evolved temperature, pollinator treatment) and non-normality in petal data, we used a glm model (generalized linear model) with a gamma distribution and a log link function. We did again two models to test for growing temperature, pollination and their interaction and evolved temperature, pollination and their interaction. Leaf data showed heteroscedasticity across pollinator treatments but was normally distributed so we chose to run two gls models to test for the effects of growing temperature, pollinator and their interaction and evolved temperature, pollinator and their interaction on aliphatic compound length. All models were followed by an anova (analysis of variance) and a post-hoc test (emmeans) to look at pairwise differences. Data was visualized using ladder plots.

We continued our analysis by examining the overall wax composition. We centered log ratio transformed (package: compositions, clr function) the proportion data prior to visualization using discriminant analysis of principal components (package: adegenet, dapc function). The optim.a.score function (package: adegenet) determined the number of used principal components. We grouped the data by pollinator treatment and made separate plots for petal and leaf data. Next, we used the clr-transformed data for permutational multivariate analyses of variance (PERMANOVA with 1000 permutations, package: vegan, adonis2 function) based on Euclidean distances. In global models including all samples across all treatments, we tested for the effects of growing temperature, evolved temperature, pollinator treatment and their interactions on petal and leaf wax composition. Pairwise comparisons across the full dataset were calculated with the pairwise.adonis2 function (package: pairwiseAdonis).

In the final step, we examined individual compounds. We first tested whether the 14 compounds occurring in petals and leaves were correlated within plants. We calculated *Pearson correlation coefficients* (cor function) between leaf and petal values, using pairwise complete observations. To identify compounds that differed significantly between temperature and pollinator treatments, we first standardized peak areas using a z-score normalization before we used the limma package to fit a linear model to each compound. The model was refined with an empirical Bayes method (package: limma, eBayes function) to improve variance estimation. Pairwise differences were extracted and P values and log-fold changes calculated and visualized using volcano plots. Significant compounds (*P* < 0.05, log2fold changes > 0.5) were highlighted in green.

## Results

After six generations of evolution under different pollinators and temperatures, *B. rapa* plants showed diverse changes in petal and leaf epicuticular waxes. We analysed total wax amounts, aliphatic chain length, and composition down to the level of individual compounds.

### Changes in Total Wax Amounts

Across all plants and treatments, leaves had significantly more epicuticular wax than petals (1.9 ± 0.07 µg/cm^2^ vs. 1.45 ± 0.08 µg/cm^2^; *Wilcox test*: *V* = 6002, *P* < 0.001). Although wax amounts varied among plants, petals and leaves showed distinct responses to temperature and pollinator treatments.

**Fig. 1:**
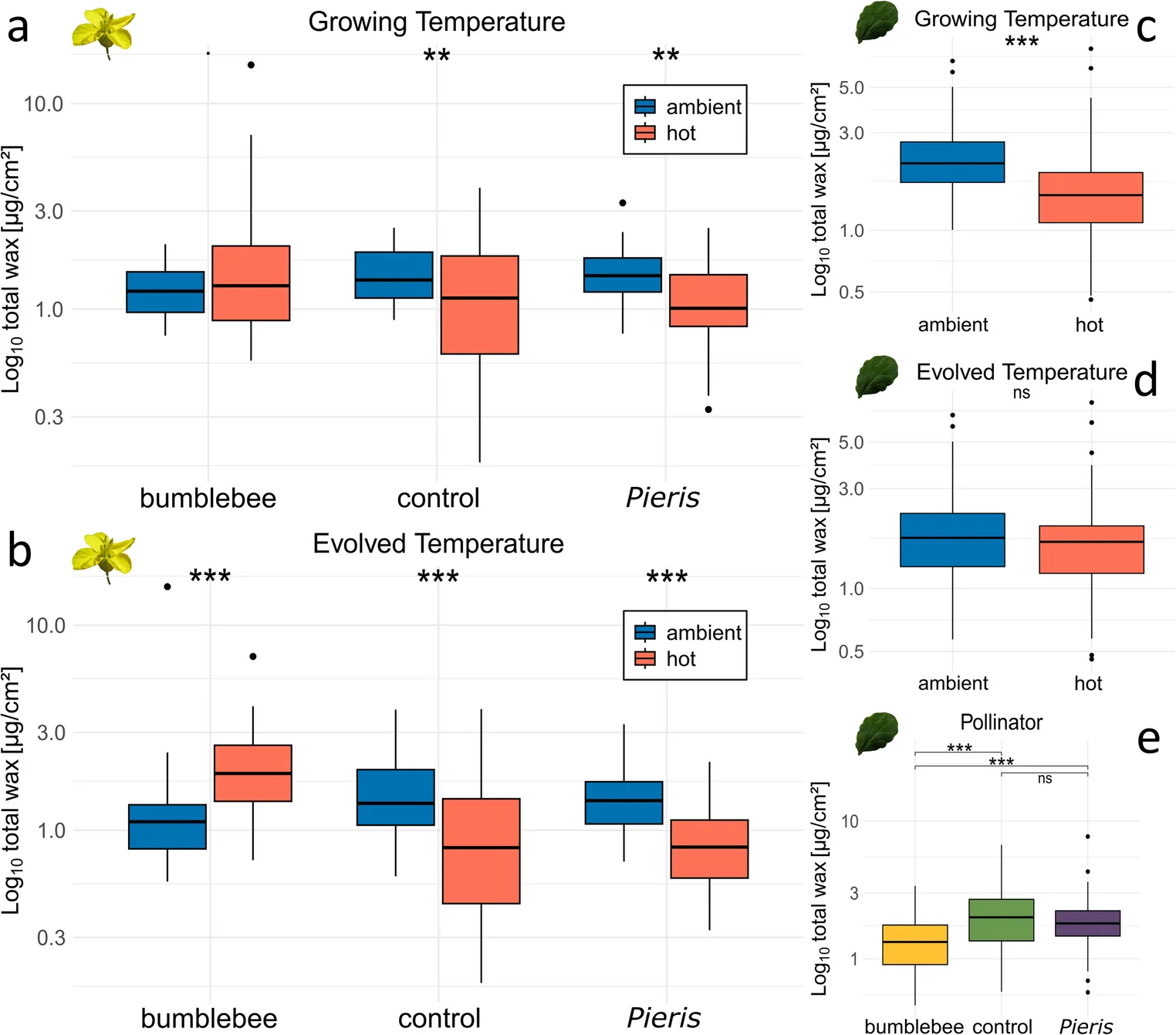
Total wax amounts in petals (**a** and **b**) and leaves (**c–e**). Panels (**a**) and (**b**) show the effects of pollinator treatment interacting with growing (**a**) and evolved (**b**) temperature on petal wax. Panels (**c**) and (**d**) show the effects of growing and evolved temperature on leaf wax, respectively, and panel (**e**) shows the effect of pollinator treatment on leaf wax. Boxplots display the median, interquartile range (25th − 75th percentile), whiskers extending to the smallest and largest values within 1.5× the interquartile range, and outliers shown as black points beyond the whiskers. Note that the y-axis is on a log scale. Asterisks above the boxes indicate statistical significance: *P* < 0.05 (*), *P* < 0.01 (**), *P* < 0.001 (***); (.) = marginally non-significant (*P* < 0.1), ns = not significant. Non-transformed values can be found in Table S1

Petal wax amount was affected by growing temperature (ambient + 16%, *F* = 6.7, *P* = 0.01) and temperature under which a plant evolved (ambient + 22%, *F* = 5.98, *P* = 0.02), but not directly by pollinator treatment (*F* = 0.052, *P* = 0.95). However, the effect of temperature was modified by pollination treatment, with bumblebees reversing the trend of wax loss in hot temperatures. We found significant interaction effects between pollinator treatment and growing temperature (Fig. 1a, F = 7.70, *P* < 0.001) and an even stronger interaction effect of pollinator treatment and temperature under which a plant evolved (Fig. 1b, F = 19.61, *P* < 0.001). This effect is mainly driven by hot bumblebee genotypes (plants that evolved in hot temperatures with bumblebee pollination), which produced 68% more wax than ambient bumblebee genotypes (*t*=-3.70, *P* < 0.001) and 148% more than hot control genotypes (*t* = 5.13, *P* < 0.001). A similar, albeit marginally non-significant, trend can be seen in bumblebee genotypes grown under hot conditions (Fig. 1a, hot bumblebee vs. ambient bumblebee: t=-1.74, *P* = 0.08).

Leaf wax amounts were mainly affected by growing temperature (Fig. 1c, ambient + 40%, *F* = 43.05, *P* < 0.001), whereas the temperature under which a plant evolved had a weaker effect (Fig. 1d, F = 1.96, *P* = 0.16). Pollinator treatment strongly influenced leaf wax amounts (Fig. 1e, F = 17.88, *P* < 0.001), with bumblebee genotypes producing ~ 37% less wax than controls, and ~ 31% less than *Pieris* genotypes (*P* < 0.001 in both comparisons). There were no interaction effects between pollinator treatment and temperature factors.

### Changes in Aliphatic Compound Length

Across all treatments, mean chain length of aliphatic compounds was ~ 7% longer in petals than in leaves (petals: 26.8 ± 0.09 carbons, leaves: 25.05 ± 0.09 carbons, *Wilcox test*: *V* = 20768, *P* < 0.001). Additionally, chain length in the two plant organs showed markedly different responses to temperature and pollinator treatments.

**Fig. 2:**
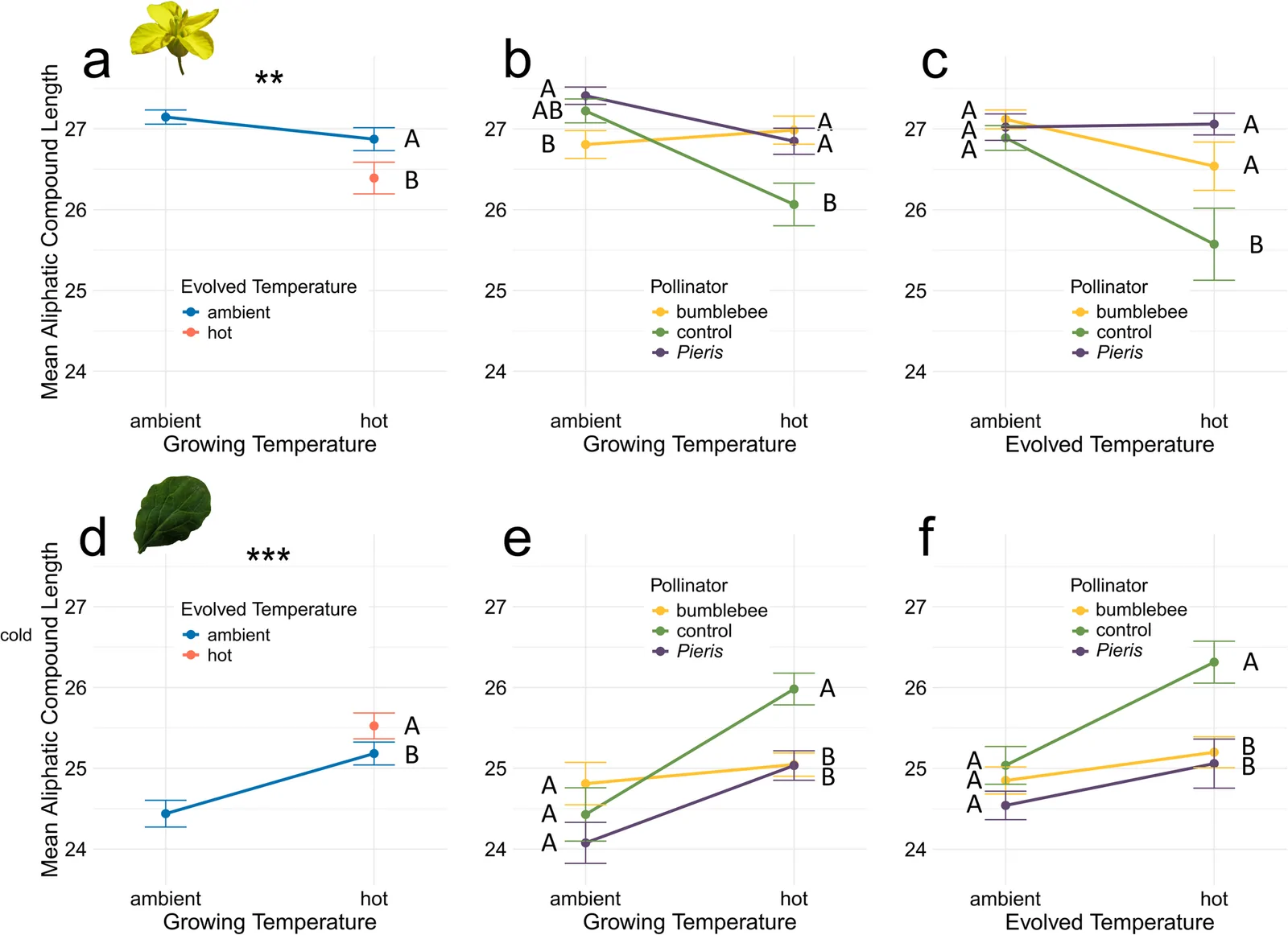
Ladder plots showing effects of different temperature and pollinator treatments on petal (**a-c**) and leaf (**d-f**) mean aliphatic compound chain length (number of carbon atoms). (**a**) and (**d**) show the effect of evolved temperature in plants exposed to different growing temperatures (each dot is the mean of 72 samples). (**b**) and (**e**) show the effect of pollinator treatment under different growing temperatures (each dot for ambient grown pollinator treatments is the mean of 24 samples, hot grown pollinator treatments are the mean of 48 samples), and (**c**) and (**f**) show the effect of pollinator treatment in plants that have evolved under different temperatures (each dot for ambient evolved pollinator treatments is the mean of 48 samples, hot grown pollinator treatments are the mean of 24 samples). Error bars show standard errors. Asterisks in plots (**a**) and (**d**) show significant differences between growing temperatures (*P* < 0.05 (*), *P* < 0.01 (**), *P* < 0.001 (***)). Letters on the left and right indicate significant differences at α = 0.05 between temperature (**a** and **d**) and pollination (**b**, **c**, **e** and **f**) genotypes

In petals, average chain length of aliphatic compounds was 2.4% longer in ambient genotypes (plants that evolved under ambient temperatures) than in hot genotypes (Fig. 2a, F = 12.01, *P* < 0.001). A similar effect was observed with growing temperature (Fig. 2a, ambient + 1.9%, *F* = 8.15, *P* = 0.005). Pollinator treatment also significantly influenced chain length (*F* = 4.65, *P* = 0.01). The effect of temperature was also influenced by pollination treatment, with insect pollinators mitigating heat- induced reductions in chain length. We found significant interaction effects between growing temperature and pollinator treatment (Fig. 2b, F = 4.6594, *P* = 0.01) and between evolved temperature and pollinator treatment (Fig. 2c, F = 4.9851, *P* = 0.008). Specifically, most insect-pollinated plants evolved or grown at hot temperatures had significantly longer aliphatic chains than hand-pollinated controls (Fig. 2b and c, Table S2). Overall, hot-grown plants produced less wax (see previous section) and shorter aliphatic chains on their petals, but insect pollination mitigated this shortening effect.

In leaves, average aliphatic chain-length was 3.6% shorter in ambient-grown plants (*F* = 19.53, *P* < 0.001) and 2.8% shorter in ambient genotypes (*F* = 11.42, *P* < 0.001) compared to hot genotypes or hot-grown plants (Fig. 2d), showing the opposite trend to petals. Pollinator treatment also had a significant effect (*F* = 5.10, *P* = 0.007), and, as in petals, temperature effects were influenced by pollination treatment. However, in leaves, insect pollination mitigated the increase in chain length. We found a significant interaction between pollinator and growing temperature (Fig. 2e, F = 4.39, *P* = 0.01) but not evolved temperature (Fig. 2f). Plants evolved with insect pollinators had shorter aliphatic chains at hot temperatures than hand-pollinated controls (Fig. 2e and f, Table S3). While chain-length increased significantly in hot-grown controls and hot control genotypes, this was not observed in hot-grown bumblebee-pollinated plants and hot bumblebee and *Pieris* genotypes. Chain length of *Pieris* genotypes increased in hot compared to ambient growing temperatures but remained similar to bee-pollinated plants. Overall, leaves had less wax (see previous section) but longer aliphatic chains in hot environments. Opposite to petals, insect pollination reduced chain length in leaves under hot conditions.

### Changing Wax Composition

**Fig. 3:**
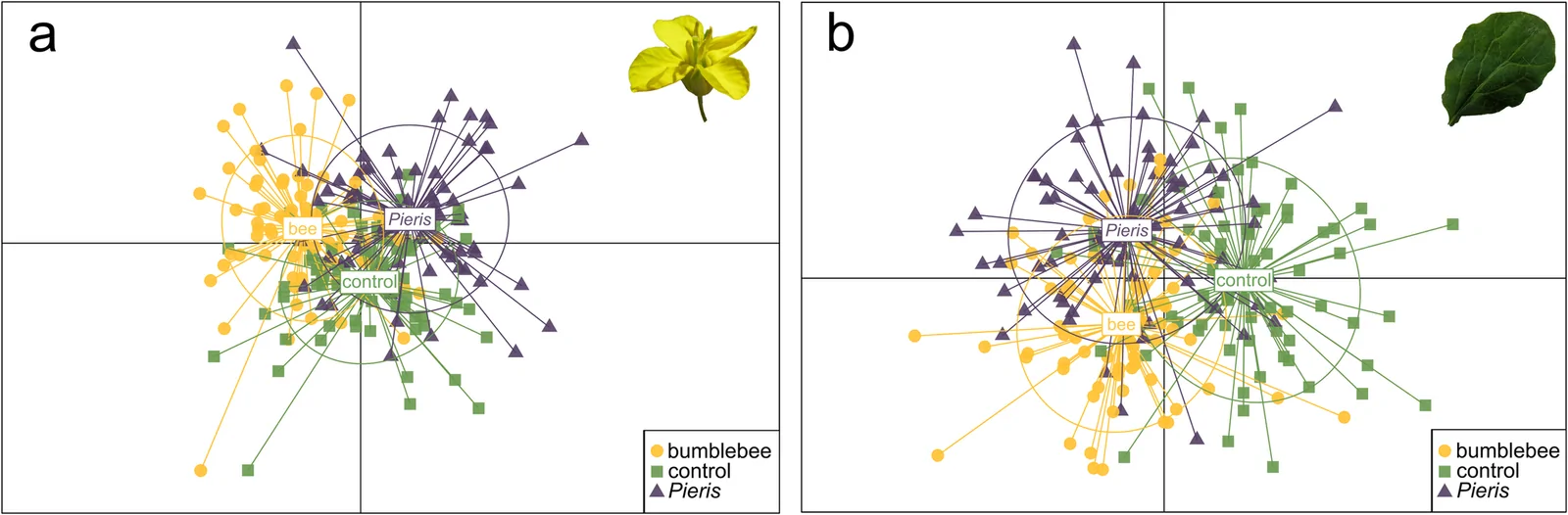
Discriminant analysis of principal components (DAPC) on petal (**a**) and leaf (**b**) wax composition in different pollinator treatments. For plot (**a**) we used 19 PCs and for (**b**) 24 PCs

In a global analysis including plants across all treatments, petal wax composition was significantly affected by pollinator treatment (PERMANOVA, *pseudo F* = 4.98, *R*^*2*^ = 0.04, *P* < 0.001), growing temperature (*pseudo F* = 10.39, *R*^*2*^ = 0.04, *P* < 0.001) and temperature under which plants evolved (*pseudo F* = 5.84, *R*^*2*^ = 0.02, *P* < 0.001). Pairwise comparisons performed on this full dataset also revealed significant differences among all pollinator treatment groups (Fig. 3a, pairwise.adonis: bee-control: *pseudo F* = 4.52, *R*^*2*^ = 0.03, *P* = 0.001, bee-*Pieris*: *pseudo F* = 4.93, *R*^*2*^ = 0.03, *P* = 0.002, control-*Pieris*: *pseudo F* = 3.78, *R*^*2*^ = 0.03, *P* = 0.006). Similar to wax amounts, wax composition showed significant pollinator x growing temperature (*pseudo F* = 2.86, *R*^*2*^ = 0.02, *P* = 0.003) and pollinator x evolved temperature (*pseudo F* = 5.35, *R*^*2*^ = 0.04, *P* < 0.001) interactions.

In leaves, like in petals, a global analysis including all plants across all treatments showed that wax composition was significantly affected by pollinator treatment (*pseudo F* = 4.07, *R*^*2*^ = 0.03, *P* < 0.001), growing temperature (*pseudo F* = 35.63, *R*^*2*^ = 0.14, *P* < 0.001), and evolutionary temperature (*pseudo F* = 3.51, *R*^*2*^ = 0.01, *P* = 0.004). Pairwise comparisons performed across all samples from all treatments showed significant differences only between insect-pollinated plants and controls, not between bumblebee and *Pieris* genotypes (Fig. 3b, pairwise.adonis, bee-control: *pseudo F* = 4.30, *R*^*2*^ = 0.03, *P* = 0.003, bee-*Pieris*: *pseudo F* = 1.38, *R*^*2*^ = 0.01, *P* = 0.186, control-*Pieris*: *pseudo F* = 4.20, *R*^*2*^ = 0.03, *P* = 0.006). Unlike petals, leaves showed no pollinator x growing temperature interaction but did show a pollinator x evolved temperature interaction (*pseudo F* = 2.12, *R*^*2*^ = 0.02, *P* = 0.03). Growing temperature had by far the strongest effect on leaf wax composition.

### Differences on the Individual Compound Level

**Fig. 4:**
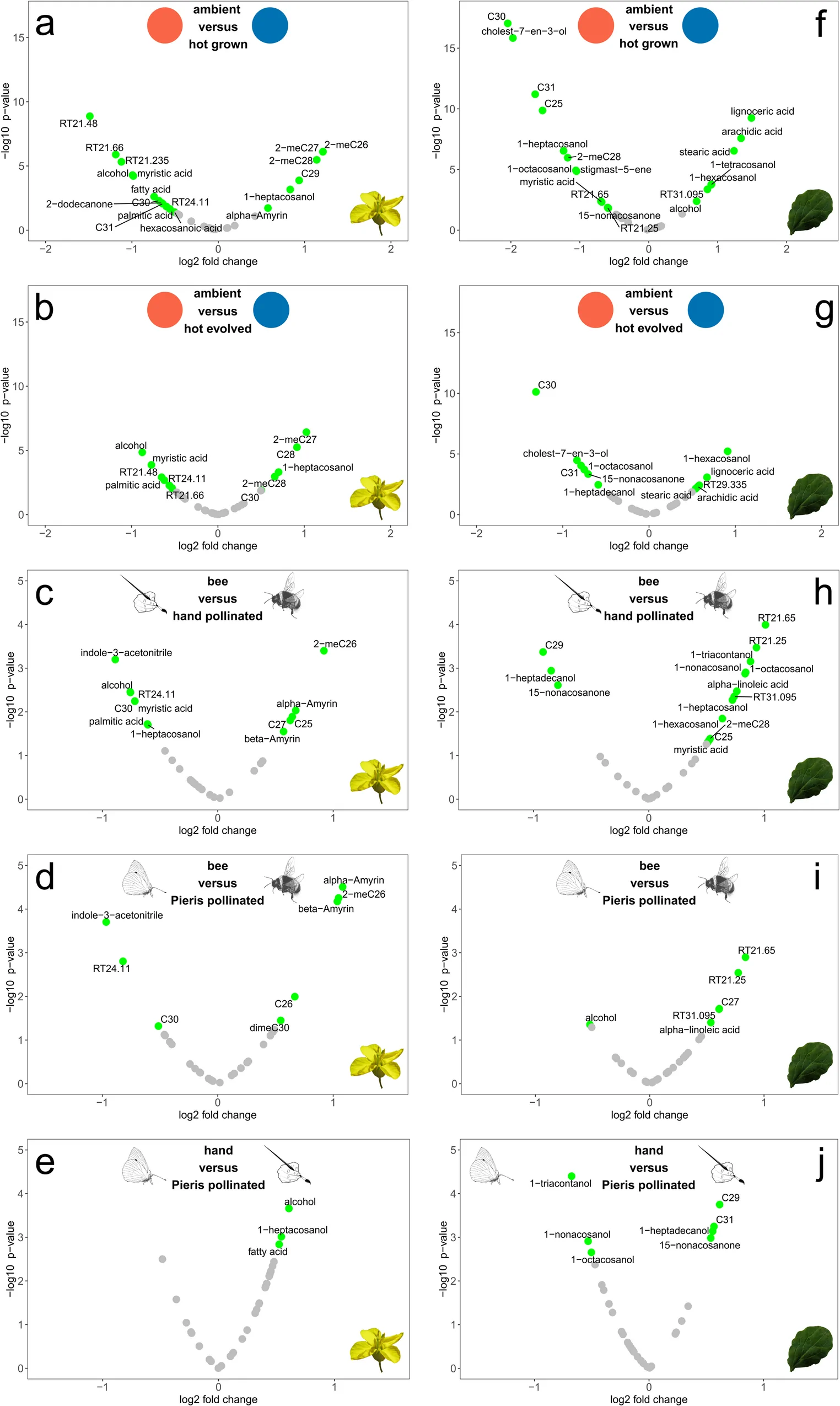
Volcano plots showing compound-level differences across petal (**a-e**) and leaf **(f-j**) wax profiles across temperature (**a**, **b**, **f** and **g**) and pollinator (**c-e** and **h-j**) treatments. Each point represents a compound and points in green indicate compounds with statistically significant differences (*P* < 0.05) and log2-fold changes > 0.5. Note: x and y-axis scales differ between temperature and pollinator comparisons to optimize visualization

We identified 30 compounds in petals and 31 in leaves (Supplementary Fig. 1), of which only 14 were shared. Abundances of shared compounds were only weakly correlated between organs (*r* = − 0.16 to 0.14). Both petals and leaves showed compound-level changes in response to temperature and pollinator treatments (Fig. 4, Supplementary Fig. 2).

In petals, we identified eight chemical classes, plus two unknown compounds. Growing temperature most strongly affected methyl-alkanes and sesquiterpenoids, with smaller changes in alcohols, ketones, and triterpenes (Supplementary Fig. 2a). Hot and ambient genotypes mainly differed in methyl-alkanes, sesquiterpenoids, alcohols, and fatty acids, while ketones and triterpenes were unchanged (Supplementary Fig. 2b). Specifically, lower growing and evolved temperatures increased levels of the homologous series of 2-methyl alkanes (2-me-C26, 2-me-C27, 2-me-C28) and 1-heptacosanol. Ambient growing temperatures also boosted the dominant linear alkane C29 (~ 37% of total wax), while plants that evolved under ambient conditions showed increases in less abundant alkanes (C28 and C30). In contrast, hot temperatures elevated several fatty acids, an unidentified alcohol, three unidentified sesquiterpenoids (RT21.235, RT21.48, RT21.66), and one unknown compound (RT24.11) (Fig. 4a and b).

In petals, pollinator treatments affected alkanes, methyl-alkanes, alcohols, alkylindoles, triterpenes, and unknowns (Supplementary Fig. 2c). Specifically, petal profiles of bumblebee genotypes show consistent changes in comparison with control and *Pieris* genotypes (Fig. 4c and d). Notably increased levels of α- and β-amyrins, as well as increases in methyl alkanes (2-me-C26, dime-C30) and linear alkanes (C25–C27). In comparison to bumblebee genotypes, *Pieris* and control genotypes shared elevated levels of indole-3-acetonitrile, along with C30 and an unknown compound (RT24.11). Control genotypes also had higher amounts of fatty acids (myristic and palmitic) and alcohols (1-heptacosanol and one unidentified). *Pieris* genotypes resembled those of hand-pollinated control genotypes (Fig. 4e), differing only in two alcohols (1-heptacosanol and an unknown) and one unidentified fatty acid, all higher in control genotypes.

In leaves, we identified ten classes plus three unknown compounds. Growing and evolutionary temperatures strongly affected composition, especially in alkanes and fatty acids, while the most abundant alcohols remained unchanged; ketones, methyl-alkanes, and sterols also showed temperature effects (Supplementary Fig. 2d and e). Specifically, hot temperatures caused a strong increase in cholest-7-en-3-ol and linear alkanes (C25, C30, C31), with a smaller rise in 15-nonacosanone. 2-me-C28, stigmast-5-ene, and two unknowns increased only in hot-grown (not evolved) plants. Alcohol and fatty acid profiles also shifted, with hot conditions favouring shorter fatty acids and longer alcohols, while ambient conditions showed the opposite pattern (Fig. 4f and g).

In leaves, pollinator treatments influenced the three dominant classes (alcohols, alkanes, fatty acids), as well as methyl-alkanes, sesquiterpenoids, and unknowns (Supplementary Fig. 2f). In comparison to control genotypes, insect pollination increased long alcohols, whereas control genotypes had more linear alkanes and 15-nonacosanone (Fig. 4h and j). Compared to control and *Pieris* genotypes, leaves of bumblebee genotypes had higher levels of long alcohols, fatty acids, 2-me-C28, C25 and C27 alkanes, plus several unidentified compounds including a sesquiterpenoid (RT_21.65)(Fig. 4h and i).

## Discussion

Epicuticular waxes on petals have received far less attention than those on leaves, as they were long assumed to play only minor roles in stress response and herbivore defense. Our study challenges this view by showing that petal and leaf waxes are both plastic and evolvable traits shaped not only by temperature but also by pollinators. In hot-genotypes and plants grown under hot conditions, petals produced less wax with shorter-chain compounds; however, insect pollination shifted wax composition away from shorter chains and, in some cases, even amplified wax production under heat. Leaves also reduced wax amounts in hot growing conditions and when they evolved under hot temperatures but, in contrast to petals, shifted towards longer-chain compounds; this increase in chain length was reduced by the effect of insect pollination. Together, these results reveal distinct but interacting wax responses in petals and leaves, highlight the role of pollinators as unexpected drivers of wax chemistry, and uncover shifts in both abundant and rare compounds with potential ecological importance. We show that floral waxes are not secondary to leaves, but rather part of a dynamic system in which temperature, pollinators, and likely other factors jointly shape plant surfaces.

Plastic plant wax responses to high temperatures have been extensively studied in crop species. In several Brassicaceae crops, higher temperatures reduced leaf epicuticular waxes (Whitecross and Armstrong 1972; Baker 1974; Armstrong and Whitecross 1976; Reed and Tukey 1982), matching our results. Yet less wax does not necessarily reduce water retention, which depends more on composition and structure (Riederer and Schreiber 2001; Grünhofer et al. 2024): nonpolar aliphatics such as alkanes reduce water loss more effectively than nonaliphatics like triterpenoids (Leide et al. 2007; Buschhaus and Jetter 2012). Plant sterols have been shown to be crucial in plant adaptation to large temperature variations (Dufourc 2008). Accordingly, under heat stress our plants increased the relative abundance of several compound classes (alkanes, ketones, methyl-alkanes, sterols), especially the C30 alkane and cholest-7-en-3-ol, despite overall wax reductions. Importantly, aliphatic chain length has been shown to be more critical for water retention than the abundance of individual compound classes (Riederer and Schneider 1990). Consistent with this, leaves grown under hot conditions produced less wax overall but were enriched in longer-chain compounds. This pattern was driven by a strong reduction in long-chain fatty acids and stable alcohol levels that shifted toward longer chains, suggesting selection for improved water retention under heat. Thus, although plasticity dominated, the consistent enrichment of potentially protective compounds, together with fatty acid losses and increased chain length in hot genotypes, points to adaptive changes in leaf wax composition under elevated temperatures.

High temperatures affected not only leaf but also petal waxes. In petals of controls and *Pieris*-genotype plants, wax loss was driven not by fatty acids (as in leaves) but by reduced methyl-alkanes and triterpenes (α/β-amyrin), while fatty acids even increased in hot-genotypes. Since amyrins weaken the intracuticular water barrier in *Arabidopsis thaliana* leaves (Buschhaus and Jetter 2012), their reduction in petals may improve water retention under heat. Notably, amyrins occurred only in petals, suggesting a flower-specific function, possibly in maintaining floral humidity (Buschhaus et al. 2015; Cheng et al. 2021), a known cue for bumblebees and hawkmoths (Von Arx et al. 2012; Harrap et al. 2021). Bumblebee genotypes consistently produced more α/β-amyrins; which also occur in bee pollen and propolis, where they may provide antimicrobial or antifungal benefits (Herrera-López et al. 2019; Yam-Puc et al. 2019; Ipav et al. 2022; Ashwathi et al. 2024), hinting bees may collect them. As sterol carrier protein inhibitors, amyrins can reduce herbivore performance (Chapman 1977; Eigenbrode and Espelie 1995; Singh et al. 2003; Kuppusamy et al. 2009; Chaudhary and Gupta 2023), but may also stimulate feeding in specialists (Tamura et al. 2004). Thus, amyrins may act at the intersection of heat adaptation, herbivore defense and pollinator interactions.

Unexpectedly, hot-bumblebee-genotype plants grown in hot conditions broke the general heat-driven wax loss patten: their petals gained wax, mainly methyl-alkanes like 2-me-C26. Insects use this compound for water balance and mate recognition (Chung et al. 2014; Wang et al. 2023), and although absent from *B. terrestris* cuticles (Jansen et al. 2016), these bees can detect related compounds. Floral methyl-alkanes may thus act as recognition cues, shaping bumblebee foraging and plant fitness. While the functional role of methyl-alkanes in plant waxes remains unclear, studies on insect cuticular hydrocarbons provide insights: methyl-alkanes have lower melting points than n-alkanes, reducing their waterproofing efficiency, especially under high temperatures (Gibbs and Pomonis 1995). However, their greater structural diversity likely enhances information content, increasing their recognition value for pollinators (Chung and Carroll 2015). The potential benefits of increased petal wax under heat for bumblebees are unclear. Although the opposite is generally the case, higher wax amounts may also improve grip, since increased wax crystal density can enhance attachment forces under certain conditions (Gorb et al. 2008), or increase flower durability during repeated visits, but this remains to be tested. Strikingly, petal wax gains coincided with reduced leaf wax, suggesting reallocation of wax from leaves to petals. Since Brassicaceae with less leaf wax are less susceptible to *Pieris* herbivory (Dickson and Eckenrode 1980; Stoner 1990; Eigenbrode and Espelie 1995), this shift might even enhance defense, though its biological relevance is uncertain. In short, bumblebees aren’t just pollinators; they may actively shape plant wax defenses, with the leaf–petal trade-off connecting heat response, herbivory, and pollinator influence.

Another trade-off emerged when comparing aliphatic chain length across treatments and plant organs. While leaf and petal wax amounts, at least in control and *Pieris* genotypes, responded similarly to hot growing and evolved temperatures, aliphatic chain length exhibited opposite trends: plants with leaves that initially had shorter aliphatic chains evolved or plastically shifted toward longer chains under heat, whereas their petals, which initially had longer chains, evolved or plastically shifted toward shorter ones. Longer compounds on petals than leaves is unusual, as a review of 57 plant species (only one from Brassicaceae, *Cakile maritima*) across seven studies (Hennig et al. 1988; Buschhaus et al. 2015; Guo and Jetter 2017; Guo et al. 2017; Cheng et al. 2019, 2021; Tunstad et al. 2024) reported generally longer aliphatics in leaves than petals. Interestingly, evolution with insect pollinators significantly altered high-temperature effects: under heat, longer leaf waxes may protect against heat and water loss but draw resources from petals, shortening chains and potentially reducing floral longevity in hand-pollinated plants, where it is fitness neutral. However, in insect-pollinated plants such changes might be costly. Consequently, they showed smaller decreases, or even increases, in petal wax length, while leaf gains were modest in response to hot growing temperatures and evolution under high temperatures. Since these effects were equally strong in *Pieris-*pollinated plants as in bumblebee-pollinated ones, we can infer that, despite the different pollination regimes, due to similar pollination efficiency but lower visitation rates in *Pieris* (Sahli and Conner 2007; Rader et al. 2009), evolution with *Pieris* pollinators also exerted strong selection on floral traits. The recurrence of this leaf–petal trade-off across both evolved and growing temperature treatments suggests an adaptive response balancing abiotic stress tolerance and pollinator-mediated selection.

These trade-offs likely extend beyond pollinators and heat to herbivores and pathogens, which also respond to epicuticular wax chemistry (Eigenbrode and Espelie 1995; Müller and Riederer 2005). Many insects use wax cues for host selection (Adati and Matsuda 1993; Lin et al. 1998; Powell et al. 1999; Morris et al. 2000; Coyle et al. 2003), and in *P. rapae* the cabbage leaf wax nonacosane (C29) deters feeding and oviposition (Ueno et al. 2025). Surprisingly, despite high C29 abundance in our *B. rapa*, six generations of *P. rapae* pollination and herbivory did not increase its concentration in leaves or petals, as might be expected if butterflies both pollinate and avoid ovipositing on high-C29 plants. Instead, *P. rapae* exposure increased leaf alcohols and maintained high petal levels of indole-3-acetonitrile, a glucosinolate breakdown product rather than a classic wax compound, that mimics *P. rapae* larval feeding and deters oviposition in Brassicaceae (de Vos et al. 2008). Pollinators modified this signal only in petals: bumblebee genotypes consistently showed lower levels than *Pieris* and controls, hinting bees may select against indole-3-acetonitrile, though direct repellence remains unproven. *Pieris* genotypes, by contrast, showed elevated leaf alcohols, echoing evidence that cabbage plants richer in primary alcohols and α/β-amyrins but poorer in linear alkanes resist specialist herbivores more effectively (Eigenbrode et al. 1991). Together, these findings suggest that the same wax traits mediating pollinator interactions may also shape plant–herbivore conflicts, highlighting the multiple selective pressures driving epicuticular wax evolution.

In conclusion, our results show that petal epicuticular waxes are not merely accessory traits: like leaf waxes, they are plastic and evolvable, responding to heat and pollinator interactions. Evolution under hot temperatures, high growing temperatures, and insect selection drive distinct yet interconnected wax adjustments in leaves and petals, affecting both abundant and rare compounds. Pollinators, particularly bumblebees, can counteract heat-driven wax loss in petals and increase compound chain length, while simultaneously reducing leaf waxes, revealing a leaf–petal trade-off between optimal wax traits. At the compound level, pollinators favoured specific molecules that may act as attractive or repellent cues. Compounds such as α/β-amyrins and methyl-alkanes may act as multifunctional mediators, potentially influencing water retention, herbivore resistance, and pollinator behaviour. Interpreting floral wax changes and adaptations in response to temperature and pollinators remains challenging, however, as the physiological bases and ecological functions of floral waxes are still poorly understood. Overall, our findings highlight epicuticular waxes as integrative traits coordinated across plant organs and shaped by both abiotic and biotic factors, with ecological and potentially agricultural implications.

## Supplementary Information

Below is the link to the electronic supplementary material.

Supplementary Material 1 (DOCX 2.34 MB)

## Data Availability

The data and code that support the ﬁndings of this study are available under the following URL/DOI: https://figshare.com/s/3dd54ccc0674d61ec8achttps://doi.org/10.6084/m9.figshare.30264571.
