# Supplementary material for: Temperature and Pollinators Drive Evolutionary and Plastic Variation in Leaf and Petal Epicuticular Waxes
Source: J Chem Ecol. 2026 Feb 13;52(1):17. doi: 10.1007/s10886-026-01691-w (PMC13649830; doi:10.1007/s10886-026-01691-w)
Supplement: Supplementary file 1 [file 10886_2026_1691_MOESM1_ESM.docx]

Supplementary Materials

Journal of chemical ecology

Temperature and Pollinators Drive Evolutionary and Plastic Variation in Leaf and Petal Epicuticular Waxes

Corinne Hertaeg*^1^, Florian P. Schiestl^1^

*^1^University of Zürich, Department of Systematic and Evolutionary Botany, Zürich, Switzerland*

*Email:* [*corinne.hertaeg@uzh.ch*](mailto:corinne.hertaeg@uzh.ch)*,* [*florian.schiestl@systbot.uzh.ch*](mailto:florian.schiestl@systbot.uzh.ch)

Figure S1. Chromatograms of GCMS runs. (a) shows the chromatogram of a representative petal pool sample and (b) from a leaf pool sample. **IS C_18_** is our internal standard octadecane. The retention times in these GC-MS runs are slightly shifted in comparison to the GC-FID runs that we used to quantify the compounds. Each number represents a different compound. **1**:unidentified alcohol RT19.81, **2**: unidentified compound RT21.25, **3**: 2-dodecanone, **4**: myristic acid, **5**: unidentified compound RT21.48, **6**: indole-3-acetonitrile, **7**: unidentified compound RT21.65, **8**: palmitic acid, **9**: unidentified compound RT24.11, **10**: unidentified fatty acid RT24.2, **11**: α-linoleic acid, **12**: stearic acid, **13**: arachidic acid, **14**: C_25_, **15**: C_26_, **16**: 2-me C_26_, **17**: C_27_, **18**: 1-tetracosanol, **19**: 2-me C_27_, **20**: 7,X-dime C_27_, **21**: C_28_, **22**: unidentified compound RT29.335, **23**: lignoceric acid, **24**: 1-pentacosanol, **25**: 2-me C_28_, **26**: C_29_, **27**: 1-hexacosanol, **28**: 2-me C_29_, **29**: 7,X-dime C_29_, **30**: C_30_, **31**: unidentified compound RT30.47, **32**: hexacosanoic acid, **33**: 1-heptacosanol, **34**: dime C_30_, **35**: 1-hexadecanol, **36**: 15-nonacosanone, **37**: C_31_, **38**: unidentified compound RT31.095, **39**: 1-octacosanol, **40**: cholest-7-en-3-ol, **41**: 1-nonacosanol, **42**: octacosanoate, **43**: 1-triacontanol, **44**: stigmast-5-ene, **45**: β-amyrin, **46**: α-amyrin. Unlabeled peaks are siloxane contaminations from the column.

Figure S2. Compound class percentages in petals (a-c) and leaves (d-f). (a) and (d) show differences between growing temperatures, (b) and (e) between evolved temperatures, and (c) and (f) between pollination treatments. Numbers after compound class labels in the x-axis indicate the number of compounds in each class. Boxplots display the median, interquartile range (25th–75th percentile), whiskers extending to the smallest and largest values within 1.5× the interquartile range, and outliers shown as black points beyond the whiskers. Significance (ns: P>0.05, *: P≤ 0.05, **: P≤ 0.01, ***: P≤ 0.001) between temperature treatments (a,b,d,e) was tested with Kruskal-Wallis tests, and between pollinator treatments (c,f) with Dunn’s tests (Bonferroni-corrected). Different letters indicate significant differences.

Table S1. Mean ± standard deviation of total wax amounts per area [µg/cm^2^] in the different treatment groups.

| **petals** | pollinator treatment | ambient greenhouse 1.43±0.06 | hot greenhouse 1.46±0.12 |  |
| --- | --- | --- | --- | --- |
|  |  |  |  |  |
| ambient evolved 1.48 ±0.11 | Hand (H) | 1.49±0.09 | 1.58±0.18 |  |
|  | Bee (B) | 1.25±0.07 | 1.67±0.63 |  |
|  | *Pieris* (P) | 1.54±0.11 | 1.36±0.10 |  |
| Hot evolved 1.38 ±0.13 | Hand (H) | - | 1.05±0.18 |  |
|  | Bee (B) | - | 2.17±0.26 |  |
|  | *Pieris* (P) | - | 0.92±0.10 |  |
| **leaves** |  | ambient greenhouse 2.38±0.13 | hot greenhouse 1.65±0.08 |  |
|  |  |  |  |  |
| ambient evolved 1.92 ±0.08 | Hand (H) | 3.04±0.28 | 1.69±0.15 |  |
|  | Bee (B) | 1.79±0.14 | 1.20±0.09 |  |
|  | *Pieris* (P) | 2.31±0.14 | 1.55±0.11 |  |
| hot evolved 1.83 ±0.14 | Hand (H) | - | 2.19±0.28 |  |
|  | Bee (B) | - | 1.29±0.13 |  |
|  | *Pieris* (P) | - | 2.01±0.27 |  |

Table S2. Pairwise contrasts of mean aliphatic compound chain length between pollinator treatments in petals of (a) plants grown at hot temperatures and (b) plants of hot genotypes. Values are based on estimated marginal means (emmeans) from separate glm models. Contrasts were Tukey-adjusted for multiple comparisons. All values are reported on the log scale.

| a | **hot growing temperature** | estimate±SE | t_(141)_ | *p* |
| --- | --- | --- | --- | --- |
|  | bee vs. control | 0.034 ±0.01 | 3.17 | 0.005 |
|  | bee vs. *Pieris* | 0.005 ±0.01 | 0.47 | 0.887 |
|  | control vs. *Pieris* | -0.03 ±0.01 | -2.7 | 0.021 |
|  |  |  |  |  |
| b | **hot evolved temperature** | estimate±SE | t_(141)_ | *p* |
|  | bee vs. control | 0.037 ±0.02 | 2.13 | 0.092 |
|  | bee vs. *Pieris* | -0.019 ±0.02 | -1.12 | 0.508 |
|  | control vs. *Pieris* | -0.057 ±0.02 | -3.24 | 0.005 |

Table S3. Pairwise contrasts of mean aliphatic compound chain length between pollinator treatments in leaves of (a) plants grown at hot temperatures and (b) plants of hot genotypes. Values are based on estimated marginal means (emmeans) from separate gls models. Contrasts were Tukey-adjusted for multiple comparisons, with degrees of freedom estimated using the Satterthwaite method.

| a | **hot growing temperature** | estimate±SE | df | t | *p* |
| --- | --- | --- | --- | --- | --- |
|  | bee vs. control | -0.935 ±0.24 | 86 | -3.84 | 0.007 |
|  | bee vs. *Pieris* | 0.012 ±0.23 | 88.9 | 0.05 | 0.999 |
|  | control vs. *Pieris* | 0.947 ±0.27 | 93.5 | 3.53 | 0.002 |
|  |  |  |  |  |  |
| b | **hot evolved temperature** | estimate±SE | df | t | *p* |
|  | bee vs. control | -1.11 ±0.32 | 42.3 | -3.46 | 0.004 |
|  | bee vs. *Pieris* | 0.14 ±0.36 | 38.7 | 0.39 | 0.92 |
|  | control vs. *Pieris* | 1.25 ±0.40 | 44.9 | 3.14 | 0.008 |
